# Typical resting-state activity of the brain requires visual input during an early sensitive period

**DOI:** 10.1093/braincomms/fcac146

**Published:** 2022-06-07

**Authors:** Katarzyna Rączy, Cordula Hölig, Maria J S Guerreiro, Sunitha Lingareddy, Ramesh Kekunnaya, Brigitte Röder

**Affiliations:** Biological Psychology and Neuropsychology, University of Hamburg, Von-Melle-Park 11, 20146 Hamburg, Germany; Biological Psychology and Neuropsychology, University of Hamburg, Von-Melle-Park 11, 20146 Hamburg, Germany; Biological Psychology and Neuropsychology, University of Hamburg, Von-Melle-Park 11, 20146 Hamburg, Germany; Biological Psychology, Department of Psychology, Carl Von Ossietzky University of Oldenburg, 26111 Oldenburg, Germany; Lucid Medical Diagnostics Banjara Hills, 500034 Hyderabad, India; Jasti V Ramanamma Children's Eye Care Center, Child Sight Institute, LV Prasad Eye Institute, 500034 Hyderabad, India; Biological Psychology and Neuropsychology, University of Hamburg, Von-Melle-Park 11, 20146 Hamburg, Germany

**Keywords:** blindness, sight recovery, congenital cataract, sensitive periods, resting-state brain activity

## Abstract

Sensory deprivation, following a total loss of one sensory modality e.g. vision, has been demonstrated to result in compensatory plasticity. It is yet not known to which extent neural changes, e.g. higher resting-state activity in visual areas (cross-modal plasticity) as a consequence of blindness, reverse, when sight is restored. Here, we used functional MRI to acquire blood oxygen level-dependent resting-state activity during an eyes open and an eyes closed state in congenital cataract-reversal individuals, developmental cataract-reversal individuals, congenitally permanently blind individuals and sighted controls. The amplitude of low frequency fluctuation of the blood oxygen level-dependent signal—a neural marker of spontaneous brain activity during rest—was analyzed. In accordance with previous reports, in normally sighted controls we observed an increase in amplitude of low-frequency fluctuation during rest with the eyes open compared with rest with eyes closed in visual association areas and in parietal cortex but a decrease in auditory and sensorimotor regions. In congenital cataract-reversal individuals, we found an increase of the amplitude of slow blood oxygen level-dependent fluctuations in visual cortex during rest with eyes open compared with rest with eyes closed too but this increase was larger in amplitude than in normally sighted controls. In contrast, congenital cataract-reversal individuals lagged a similar increase in parietal regions and did not show the typical decrease of amplitude of low-frequency fluctuation in auditory cortex. Congenitally blind individuals displayed an overall higher amplitude in slow blood oxygen level-dependent fluctuations in visual cortex compared with sighted individuals and compared with congenital cataract-reversal individuals in the eyes closed condition. Higher amplitude of low-frequency fluctuation in visual cortex of congenital cataract-reversal individuals than in normally sighted controls during eyes open might indicate an altered excitatory–inhibitory balance of visual neural circuits. By contrast, the lower parietal increase and the missing downregulation in auditory regions suggest a reduced influence of the visual system on multisensory and the other sensory systems after restoring sight in congenitally blind individuals. These results demonstrate a crucial dependence of visual and multisensory neural system functioning on visual experience during a sensitive phase in human brain development.

## Teaser

Rączy et al. report altered slow blood oxygen level-dependent fluctuations during rest in sight recovery individuals indicating that the typical excitatory–inhibitory balance of visual cortex, as well as the interaction of the visual system with other sensory and multisensory systems require visual input during a sensitive period.

## Graphical Abstract

**Graphical abstract:**
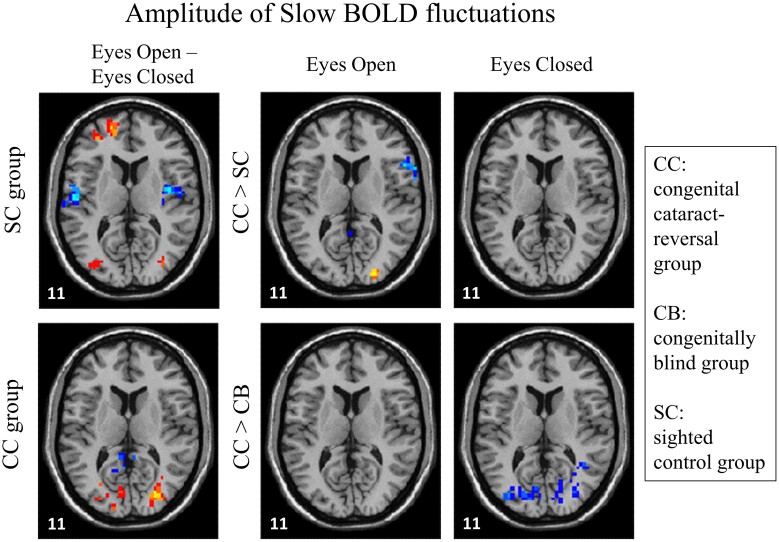
Amplitude of Slow BOLD fluctuations Eyes Open – Eyes Closed Eyes Open Eyes Closed SC group CC group CC > SC CC > CB CC: congenital cataract-reversal group CB: congenitally blind group SC: sighted control group.

## Introduction

A number of studies have demonstrated that sensory deprivation, due to e.g. blindness, results in functional and structural changes of the brain related to intra- and cross-modal plasticity.^1,2^ While intra-modal plasticity comprises reorganizations of neural systems predominantly linked to the intact sensory modalities, e.g. the auditory system,^3–5^ cross-modal plasticity refers to an activation of neural circuits primarily associated with the deprived sensory modality, e.g. the visual cortex, by input of the intact sensory modalities (e.g. auditory input). A higher activation of visual cortex in permanently blind humans during rest^6,7^ has been interpreted as a higher excitatory–inhibitory (E/I) balance allowing for cross-modal activation.^8^ Both intra- and cross-modal plasticity have been linked to compensatory performance in blind humans.^9^

The question has been raised whether adaptations to blindness, in particular cross-modal plasticity interfere with functional recovery when vision is restored by preventing the functional tuning of visual areas for visual processing.^2,9,10^ Individuals, who due to the presence of bilateral dense congenital cataracts did not experience any patterned vision after birth, have been shown to suffer persisting visual deficits in multiple visual functions.^11–16^ Yet it is unknown whether cross-modal plasticity retracts following sight restoration and whether typical visual processing circuits emerge.^2,15,17^ There is some evidence suggesting a larger influence of the auditory system on the visual system in sight recovery individuals with short deprivation epochs using brain imaging^18–21^ and behavioural measures,^22^ whereas electrophysiological studies have not yet found analogous evidence.^15^ Recent results in the deaf cat have argued against cross-modal plasticity limiting auditory recovery.^23,24^ In two brain imaging studies in short deprived congenital cataract-reversal individuals cross-modal activation of their visual cortex elicited by auditory stimulation^18,19^ was rather weak compared with the typical cross-modal activation observed in permanently blind humans.^25^ However, longer deprivation periods might cause more extensive and persisting cross-modal plasticity. It has been suggested that what causes persisting visual deficits in visual functions^26,27^ is the lack of an experience dependent functional and structural tuning^28,29^ of genuine visual processing. Based on this hypothesis we would predict altered resting-state activity in visual brain regions after restoring sight in individuals born without pattern vision. Such a lack of reversibility of typical resting-state activity profiles would be evidence for a sensitive period for typical brain development.^2,30^

Resting brain activity is considered as crucial scaffold for task-related processing. For example, it has been found that resting-state connectivity in the visual cortex of awake ferrets mimics the typical visual elicited brain activity.^31^ In humans, Biswal et al.^32^ demonstrated that low frequency blood oxygen level-dependent (BOLD) fluctuations (0.01–0.08 Hz) within the sensorimotor network during rest mirrored typical sensorimotor activity during a finger tapping task indicating that resting-state BOLD fluctuations correlate across functionally related, even spatially remote brain regions similarly as during task performance.^33^ Resting-state BOLD connectivity often reflects structural connectivity as assessed with diffusion spectrum imaging in humans^34^ and as demonstrated by combining non-invasive functional magnetic resonance imaging (fMRI) and invasive anatomical retrograde tracing methods in monkeys.^35^ Resting-state connectivity, does not, however, provide information about the level of BOLD signal change. In contrast, the amplitude of low-frequency fluctuation (ALFF)^36^ indicates activity in the low frequency range (0.01–0.08 Hz) of the BOLD response. ALFF reflects the strength of low frequency oscillations (LFOs) and is considered as a good estimate of the overall activity in neural circuits.^36,37^

ALFF has been shown to indicate changes in overall neural activity in psychiatric and neurological populations^37–43^ and in blind humans^44^ as compared with the control group. In healthy sighted subjects, ALFF has been compared between eyes open (EO) and eyes closed (EC) resting states. Typically, during EO ALFF is higher than during EC in higher visual cortices (i.e. middle occipital gyrus) and in orbital frontal cortex, whereas ALFF was found to be decreased in the bilateral pre- and postcentral gyrus, as well as in temporal and insula regions, the thalamus^44–47^ and in most studies in primary visual cortex.^44,46,47^

Simultaneous EEG-fMRI studies reported a negative correlation of the ALFF and EEG alpha oscillatory activity.^48,49^ During rest, alpha activity is higher with EC than with EO.^50,51^ Alpha band activity has been suggested to reflect a neural mechanism important for the control of the E/I balance of neural circuits to guarantee efficient processing: During task processing alpha band power is high for neural circuits not engaged in task processing but low for task relevant neural systems^52^ resulting in an improved signal-to-noise ratio in neural networks.

Posterior alpha oscillatory activity was found to be significantly reduced in congenital cataract-reversal individuals as compared with developmental cataract-reversal individuals (that is, individuals with a history of late onset cataracts) and typically sighted controls.^53^ The authors speculated that the reduced alpha activity in congenital cataract-reversal individuals was due to a reduction of inhibitory mechanisms which seem to be elaborated during sensitive periods.^29^ In fact, reduced posterior alpha activity is a typical characteristic of the EEG of permanently blind humans.^54–57^ Moreover, two magnetoencephalography studies later reported enhanced gamma activity in congenitally permanently blind individuals compared with typically sighted controls.^58,59^ Recent studies have suggested that alpha and gamma oscillatory activity indicate antagonistic mechanisms with alpha oscillations controlling gamma oscillatory activity.^60,61^ Interestingly, in monkeys, it has been observed that slow BOLD fluctuations positively correlate with high frequency local field potentials (LFPs) in the gamma range particularly in an EC condition.^62^

Based on the group differences in oscillatory brain activity (lower alpha activity in both congenital cataract-reversal and congenitally blind individuals, higher gamma activity in congenitally blind humans), we hypothesized that ALFF is enhanced in congenital cataract-reversal individuals, as well as in congenitally blind individuals compared with typically sighted controls in occipital brain regions. However, resting-state brain activity has not yet been investigated in congenital cataract-reversal individuals. Since resting-state activity builds the foundation of stimulus driven activity and might reflect a reinforcing process for the existing functional connectivity,^62,63^ assessing the amplitude of low frequency oscillations of the BOLD signal in congenital cataract-reversal individuals compared on the one hand to congenitally permanently blind humans and on the other hand, to typically sighted controls allows investigating the degree of functional recovery of visual neural circuit functioning following sight restoration. Hence, the present study investigated the question of whether there is a sensitive period for the development of typical resting-state activity in humans and thus a retraction of cross-modal plasticity as observed in congenitally blind humans.

Resting-state fMRI (rs-fMRI) was recorded both during EO and EC in four groups: (i) congenital cataract-reversal individuals (CC group) who had experienced visual deprivation since birth for up to 18 years due to bilateral dense congenital cataracts, (ii) congenitally permanently blind individuals (CB group) to indicate the adaptation of resting-state activity to congenital blindness, (iii) developmental cataract-reversal individuals (DC group) who had developed cataracts later during childhood to serve as a control for visual impairments and other effects related to a history of cataracts and cataract removal surgery and (iv) typical sighted controls (SC group).

Low-frequency oscillations (range: 0.01–0.08 Hz) of the BOLD signal were assessed as described in Yu-Feng et al.^36^ As aforementioned, we hypothesized to find overall higher ALFF in visual areas of both CC and CB individuals compared with the SC group. As previous studies have revealed retracted cross-modal activity in the CC group^19,64^ compared with what has been typically found in CB individuals,^2,65^ we predicted on the one hand that ALFF is overall lower in the CC group than in the CB group in early visual areas (including striate and extrastriate brain regions) and on the other hand that ALFF is modulated by EO versus EC in the CC group similarly as seen in normally sighted individuals. In particular, we hypothesized to replicate the typical decrease of ALFF in the EO compared with the EC condition in early visual cortex (striate cortex) and an increase in extrastriate and higher visual areas in both the CC and the SC group. Previous research on resting-state functional connectivity in CB individuals indicated lower connectivity between visual and both sensorimotor and auditory areas^66^ in particular when compared with sighted individuals with EC.^67^ Moreover, several studies have demonstrated a positive correlation between ALFF and functional resting-state connectivity.^68,69^ Thus, assuming incomplete recovery in CC individuals, we predicted a lower decrease of ALFF for the EO compared with the EC condition in auditory and sensorimotor areas compared with the SC group. We expected the CC versus SC group difference to be specific for the CC group, that is, we predicted to not finding the same pattern of results for the DC versus SC group comparison.

## Materials and methods

### Participants

All participants were recruited at the LV Prasad Eye Institute (LVPEI) and from the local community of Hyderabad (India). Four groups were included: congenital cataract-reversal individuals (CC group), developmental cataract-reversal individuals (DC group), congenitally permanently blind individuals (CB group) and sighted controls (SC group).

The original CC group consisted of 22 individuals. Three of them were excluded from the final analysis; one because of insufficient data due to premature termination of the scanning session (C7), a second due to a deprivation period of <3 months (C12) and in a third participant (C13) it turned out later that cataracts had not been dense. The final CC group consisted of 19 individuals (nine men, 10 women, mean age: 16.9 years, range: 6–36 years, mean age at surgery: 67.9 months, range: 3–216 months; mean time since surgery: 138.9 months, range: 6–412 months). Mean Logarithm of the Minimum Angle of Resolution (logMAR) visual acuity in the better eye (based on the most recent entry in the medical records) was 0.86 (range: 0.30–1.80). One participant’s visual acuity could not be measured with the letter charts. Thus visual acuity was assessed with the maximal distance at which the participant was able to count fingers, which in this particular case was 1 m (which was translated to 1.80 logMAR). We only included participants with a history of bilateral dense congenital cataracts confirmed based on the information available in the medical records. In the classification process, additionally to the clinical diagnosis, we considered factors such as presence of sensory nystagmus and strabismus, absence of fundus view before surgery, and a positive family history. For detailed information about the participants, see Table 1.

**Table 1:** Clinical and demographic information for congenital cataract-reversal participants

Participant	Sex	Age (years)	Cataract onset	Pre-surgery visual acuity in the better eye (logMAR)	Age at surgery (months)	Visual acuity in the better eye at testing (logMAR)	Additional details
CC1	M	24	Congenital	N/A	5	0.80	Nystagmus, esotropia
CC2	M	36	Congenital	N/A	24	0.40	Nystagmus, esotropia
CC3	M	9	Congenital	CF at 3 m^a^	84	0.80	Nystagmus,
CC4	F	6	Congenital	PL+, PR+	48	0.90	Nystagmus,
CC5	F	18	Congenital	CFCF^a^	192	CF at 1 m (1.80 logMAR)	Nystagmus, exotropia, microcornea -
CC6	M	32	Congenital	N/A	72	1.30	Nystagmus,
CC7*	M	11	Congenital	PL+, PR+	61	0.78	Nystagmus, esotropia
CC8	F	28	Congenital	CFCF	216	1.10	Nystagmus, esotropia
CC9	M	11	Congenital	FFL	5	0.90	Nystagmus, esotropia
CC10	M	28	Congenital	N/A	168	1.00	Nystagmus,
CC11	M	13	Congenital	FFL	15	0.30	Nystagmus, exotropia
CC12*	M	8	Congenital	FFL	1	0.20	Pseudophakia^b^
CC13*	M	30	Congenital	1.10^a^	216	1.30	Pseudophakia, Nystagmus
CC14	F	26	Congenital	N/A	8	0.80	Exotropia^b^
CC15	F	10	Congenital	FFL	42	0.40	Nystagmus, exotropia
CC16	F	7	Congenital	FFL	3	0.80	Nystagmus, esotropia
CC17	F	17	Congenital	CFCF^a^	123	1.48	Nystagmus^c^
CC18	F	11	Congenital	CF at 1 m	127	1.30	Nystagmus
CC19	M	8	Congenital	FFL^a^	64	0.40	Nystagmus, esotropia
CC20	F	18	Congenital	FFL at 1 m	25	0.48	Nystagmus, esotropia, microcornea
CC21	M	13	Congenital	CF at 0.5 m	64	0.74	Nystagmus, esotropia
CC22	F	6	Congenital	No FFL	5	0.60	Nystagmus, esotropia

*Note.* M = male; F = female; N/A = not available; CF = counting fingers at *n* metres; PL+ = able to perceive light; CFCF = counting fingers close to face; FFL = fixing and following light; PR+ = able to report the location of light.

^a^ partially absorbed cataracts

^b^ the presence of nystagmus was not reported in the medical file

^c^ operated only in one eye

*excluded participants

The original DC group consisted of 16 individuals. Five participants (D12–16) were excluded from the final analysis. We were not able to come to an unambiguous classification and thus refrained from including these participants in either the DC group or the CC group. The final DC group consisted of 11 individuals (eight men, three women, mean age: 15.8 years, range: 9–43 years, mean age at surgery: 157.3 months, range: 84–484 months; mean time since surgery: 36.6 months, range: 7–60 months; mean logMAR visual acuity, according to the most recent entry in the medical records: 0.37, range: 0.10–1.78; For detailed information about the participants, see Table 2). Note that though the age at surgery is known in DC individuals, the exact age at cataract onset is hard to define, since developmental cataracts typically gradually emerge. Furthermore, developmental cataracts were not necessarily dense as in the CC group. The DC group served as a control for visual impairments and other effects related to a history of cataracts and cataract removal surgery.

**Table 2:** Clinical and demographic information for developmental cataract-reversal participants

Participant	Sex	Age (years)	Cataract onset	Pre-surgery visual acuity in the better eye (logMAR)	Age at surgery (months)	Visual acuity in the better eye at testing (logMAR)	Additional details
DC1	M	13	Developmental	PL+, PR+	108	1.78	Nystagmus, exotropia OU
DC2	M	13	Developmental	0.80	150	0.10	—
DC3	F	43	Age-related^b^	0.60	484^c^	0.30	—
DC4	M	12	Congenital	0.80	142	0.60	—
DC5	M	9	Developmental	1.04	86	0.30	—
DC6	M	9	Developmental	0.90	84	0.10	—
DC7	M	17	Developmental	0.40	150	0.10	—
DC8	M	13	Developmental	1.00	96	0.20	Exotropia
DC9	M	11	Developmental	0.48	100	0.30	—
DC10	F	13	Developmental	0.50	122	0.10	—
DC11	F	21	Developmental	0.40	208	0.20	—
DC12*	F	34	Congenital	1.48^a^	376	1.10	Nystagmus, exotropia
DC13*	M	32	Developmental	CF at 2 m	108	1.30	Nystagmus, iris coloboma OU
DC14*	M	6	Congenital	1.30	67	0.80	Nystagmus
DC15*	M	N/A	Congenital	N/A	N/A	0.40	—
DC16*	F	N/A	Congenital	CF at 2 m^a^	183	1.30	Exotropia

*Note.* M = male; F = female; OU = both eyes; N/A = not available; CF = counting fingers at *n* metres; PL+ = able to perceive light; PR+ = able to report the location of light.

^a^ partially absorbed cataracts

^b^ cataracts developed after the age of 12

^c^ operated only in one eye

*excluded participants

The CB group consisted of 12 congenitally blind individuals. Three participants were excluded from the final analysis; one (CB6) due to the lack of the EO condition, a second (CB11) due to a central cause of blindness and a third (CB12) due to non-congenital blindness. The final CB group consisted of nine individuals (six men, three women, mean age: 20 years, range: 9–39 years). For detailed information about the participants, see Table 3.

**Table 3:** Clinical and demographic information for congenitally blind participants

Participant	Sex	Age (years)	Cause of blindness/diagnosis	Blindness onset	Visual acuity
CB1	M	39	Microphthalmia OU	Congenital	N/A
CB2	M	9	Lebers congenital amaurosis OU	Congenital	FFL
CB3	M	21	Microphthalmos OU, Microcornea OU,	Congenital	NLP
CB4	F	19	Microphthalmos OU	Congenital	PL+
CB5	M	17	Phthisis Bulbi OD, Anterior Staphyloma OS	Congenital	PL+
CB6*	M	16	Microphthalmos OD, Anophthalmos OS	Congenital	PL+
CB7	F	19	Anterior staphyloma OD, Phthisis Bulbi OS	Congenital	PL+
CB8	M	23	Corneal scar OU, Microphthalmia OU	Congenital	CFCF
CB9	M	16	Microphthalmia OU, band-shaped Keratopathy OU	Congenital	PL+, PR+
CB10	F	17	Lebers Congenital Amaurosis OU	Congenital	NLP
CB11*	M	N/A	Cortical lesions	Congenital	PL+
CB12*	N/A	N/A	N/A	Late-onset	N/A

*Note.* M = male; F = female; OU = both eyes; OD = right eye; OS = left eye; N/A = not available; FFL = fixing and following light; NLP = no light perception; PL+ = able to perceive light; PR+ = able to report the location of light; CFCF = counting fingers close to face.

*excluded participants

The SC group comprised 28 individuals (19 men and nine women, mean age: 21.6 years, range: 6–56 years). Nineteen of 28 sighted controls were matched in age and sex with the CC group (11 men and 8 women, mean age: 19.8 years, range: 6–41 years). Eleven of 28 sighted controls were matched in age and sex with the DC group (eight men and three women, mean age: 18.6 years, range: 10–42 years). Nine of 28 sighted controls were matched in age and sex with the CB group (six men and three women, mean age: 20.8 years, range: 10–41 years). All sighted control participants had normal or corrected to normal vision.

All participants or their legal guardians (in case of minors) provided written informed consent and an assent (in case of minors) prior to taking part in the study. The subjects’ consent was obtained according to the Declaration of Helsinki. Participants and in case of minors, their legal guardians received a small compensation for the time of participation (e.g. lost wages) and for other expenses such as travel costs.

The study was approved by the Local Ethics Board of the Faculty of Psychology and Movement Sciences, University of Hamburg, Germany, the Ethics Board of the German Psychological Society, as well as the Institutional Ethical Review Board of the LVPEI.

### MRI acquisition

Data were acquired at a radiology clinic (Lucid Medical Diagnostics Banjara Hills, Hyderabad, India), with a 1.5 T GE Signa HDxt scanner. Resting-state fMRI scans were collected employing a gradient-echo echo-planar imaging (EP/GR) sequence. An 8-channel head coil was used [flip angle = 90°; repetition time (TR) = 2000 ms; echo time (TE) = 30 ms, Field Of View = 220 × 220 mm; 64 × 64 matrix]. TRs varied slightly among the participants (range: 1950–2300) due to the participant's head size, body weight and height. This TR adjustment was built into the available GE protocol. Thirty-two (or in one subject 38, in a second—34 and in three subjects—33) interleaved axial slices (thickness 3 mm; in-plane resolution = 3.4 × 3.4 mm^2^, interslice gap = 4 mm) in ascending order were acquired. Anatomical T_1_-weighted images, using 3D-spoiled gradient recalled (3D-SPGR) sequence [TR = 14.7 ms (range: 14.64–15.03); TE = 6.62 ms (range: 6.60–6.76), FA = 15°; on average 187 axial slices (range: 172–196 slices); voxel dimensions = 0.8 × 0.4297 × 0.4297 mm; matrix size = 512 × 512; inversion time = 500 ms, slice thickness = 1.6 mm, slice gap = −0.8 mm] were additionally acquired for each subject.

### MRI acquisition—procedure

Two runs of rs-fMRI were acquired for each participant, one with EO and one with EC. Each run lasted for 8.53 min: 45 participants started with the EO condition and the remaining 22 with the EC condition. Note that counterbalancing of the conditions across groups was not perfect due to miscommunication in a clinical setting: 12 CC individuals started with the EO and seven with the EC, eight DC individuals started with the EO and three with the EC, seven CB individuals started with the EO and two with the EC, 18 SC individuals started with the EO and 10 with the EC. During the rs-fMRI scanning, in both conditions, the participants were asked to lay as still as possible, to not think about anything in particular and to not fall asleep. In the EO condition, the subjects were asked to keep their eyes open and in the EC condition to keep their eyes closed throughout the whole run. The scanner room was kept dimly lit during scanning.

### Data preprocessing

Data preprocessing and analysis of ALFF was performed using the DPARSF software of DPABI V4.3.^70^ DPABI is a toolbox for Data Processing and Analysis of Brain Imaging based on SPM12 and REST implemented in MATLAB (MathWorks, Natick, MA, USA).

First, basic screening of images (visual inspection) for each participant was performed to check for image quality. Exclusion criteria were signal loss due to susceptibility artefacts and cut off slices. No images were excluded due to this procedure. Then, all time points were transformed from echo-planar imaging (EPI) Digital Imaging and Communications in Medicine (DICOM) to Neuroimaging Informatics Technology Initiative (NIFTI). The first 10 time points were removed for signal stability. Next, a standard preprocessing pipeline was applied: All the acquired functional volumes were aligned to the first slice for EPI distortion and slice acquisition time (slice timing). The functional volumes were subsequently spatially realigned (using rigid body transformations to correct for head movements), spatially normalized to the standard adult East Asian template (MNI space), and smoothed with a 4 mm full width at half maximum Gaussian kernel. In the next step, linear trends were removed from the time series (detrend) using polynomial regressors. Structural images were segmented into grey matter, white matter (WM) and CSF. Subsequently, nuisance covariates were regressed out: Friston 24 head motion parameters, CSF and WM signals. The use of full nuisance regression including polynomial detrending in ALFF data optimizes the group-level analysis.^71^ In the next step, the time series for each voxel were filtered (band-pass filtering: 0.01–0.08 Hz),^32,46^ to remove the effects of very-low-frequency drifts and high frequency noise. For a given voxel, a fast Fourier transform (FFT) (parameters: taper percent = 0, FFT length = shortest)^72^ was used to convert the filtered time series to the frequency domain to obtain the power spectrum. The power spectrum was then square-rooted and averaged across the frequency band of 0.01–0.08 Hz at each voxel, which represents ALFF. Finally, the amplitudes (beta scores) of subject-level maps were transformed into *Z*-scores to create standardized subject-level maps for each participant in the EO and in the EC condition for the statistical analysis.

### Data analysis

**Figure 1:**
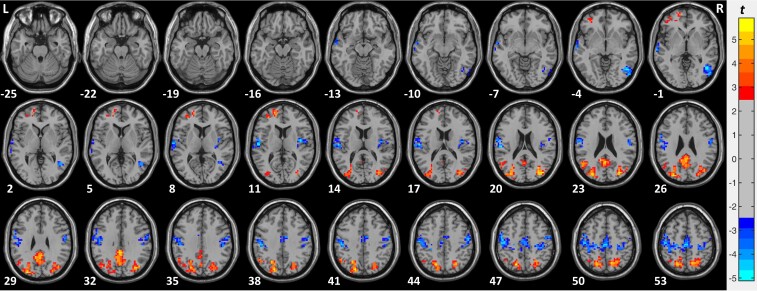
**ALFF in the EO compared with the EC condition in sighted control individuals (SC group, *n* = 28).** Paired *t*-test results of the ALFF comparing the EO and the EC condition in the group of sighted control individuals (SC group, *n* = 28). The red colours denote voxels with significantly higher amplitude in the EO compared with the EC condition and the blue colours denote voxels with significantly lower amplitude in the EO compared with the EC condition. Significant clusters are shown after GRF correction for multiple spatial comparisons (voxel-wise *P* < 0.01, cluster-wise *P* < 0.05, corrected).

To determine whether we were able to replicate previous results assessed with ALFF in the EO compared with the EC condition in typically sighted individuals,^45–47,73^ a voxel-wise paired *t*-test was carried out in the group of 28 typically sighted individuals comparing the EO and the EC condition (Fig. 1; Supplementary Table 1). The statistical map was corrected for multiple comparisons using a Gaussian Random Field (GRF) correction (voxel-wise *P* < 0.01, cluster-wise *P* < 0.05, corrected).^74^

**Figure 2:**
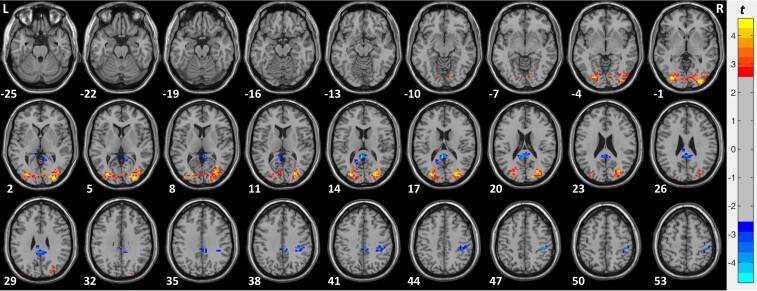
**ALFF in the EO compared with the EC condition in CC individuals (CC group, *n* = 19).** Paired *t*-test results of the ALFF comparing the EO and the EC condition in the group of congenital cataract-reversal individuals (CC group, *n* = 19). The red colours denote voxels with significantly higher amplitude in the EO compared with the EC condition and the blue colours denote voxels with significantly lower amplitude in the EO compared with the EC condition. Significant clusters are shown after GRF correction for multiple spatial comparisons (voxel-wise *P* < 0.01, cluster-wise *P* < 0.05, corrected).

To determine the brain regions with significantly higher and lower ALFF between the EO compared with the EC condition in each of the tested groups, voxel-wise paired *t*-tests were separately calculated for: (i) the SC group matched in sex and age to the CC group, (ii) the CC group, (iii) the CB group and (iv) the DC group (Fig. 2; Supplementary Figs. 3A–C; Supplementary Table 2). The statistical maps were corrected for multiple comparisons using a GRF correction (voxel-wise *P* < 0.01, cluster-wise *P* < 0.05, corrected).^74^

**Figure 3:**
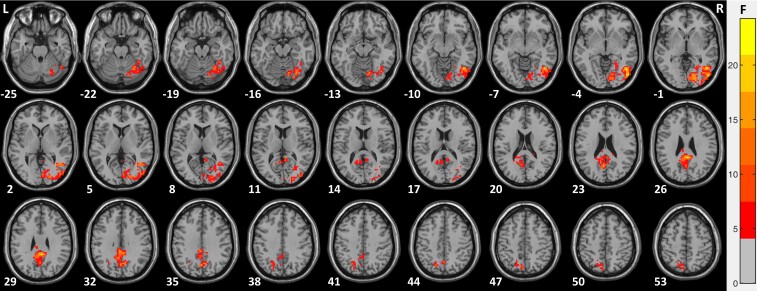
**Group differences in ALFF in the EO compared with the EC condition in CC individuals compared with the SC group (*n* = 19).** Using standardized ALFF i.e. *Z*-scores, a mixed 2 × 2 model (group x condition) was carried out for congenital cataract-reversal individuals (CC) versus the SC group (*n* = 19). Regions with significant interaction effects are shown after GRF correction for multiple spatial comparisons (voxel-wise *P* < 0.05, cluster-wise *P* < 0.025, corrected).

To assess group differences in ALFF between the EO compared with the EC condition, a 2 × 2 mixed effect model on standardized ALFF maps (*Z*-scores) was carried out. Three group models were run: (i) CC group versus SC group; (ii) DC group versus SC group; (iii) CB group versus SC group (Fig. 3; Table 4; Supplementary Figs. 1A and B). Note that in a mixed effect model, we matched typically sighted individuals approximately in age and sex to the participants of each tested group (see *Participants* section). All interaction maps were corrected for multiple comparisons using the GRF correction with a threshold of voxel-wise *P* < 0.05, cluster-wise *P* < 0.025, corrected.^75–77^

**Table 4:** Group differences in ALFF in the EO compared with the EC condition

Hemisphere	Brain region	Brodmann area	Cluster size	Peak voxel MNI coordinates			Peak voxel F-value
				x	y	z	
ALFF in the CC group versus the SC group (*n* = 19) in the EO versus the EC condition							
R	Middle Occipital Gyrus/Lingual Gyrus/Calcarine Gyrus/Fusiform Gyrus	17, 18, 19, 37	482	48	−64	−4	21.50
R	Precuneus/Cingulate Gyrus	7, 23	306	3	−44	28	18.98
ALFF in the DC group versus the SC group (*n* = 11) in the EO versus the EC condition							
L	Precuneus/Cingulate Gyrus	7, 23	221	−4	−61	48	16.73
ALFF in the CB group versus the SC group (*n* = 9) in the EO versus the EC condition							
L	Superior Parietal Gyrus/Precuneus	7	781	−25	−64	48	29.82
R	Superior Frontal Gyrus/Middle Frontal Gyrus/Medial Frontal Gyrus	8, 9, 46	674	17	49	16	28.24

Clusters showing group differences in ALFF between the EO and the EC condition at *P* < 0.05 voxel-wise and *P* < 0.025 cluster-wise after GRF correction for multiple comparisons. MNI coordinates and F-values are derived from the peak voxel of the cluster. EO = eyes open. EC = eyes closed. MNI = Montreal Neurological Institute coordinates system. L = left. R = right.

**Figure 4:**
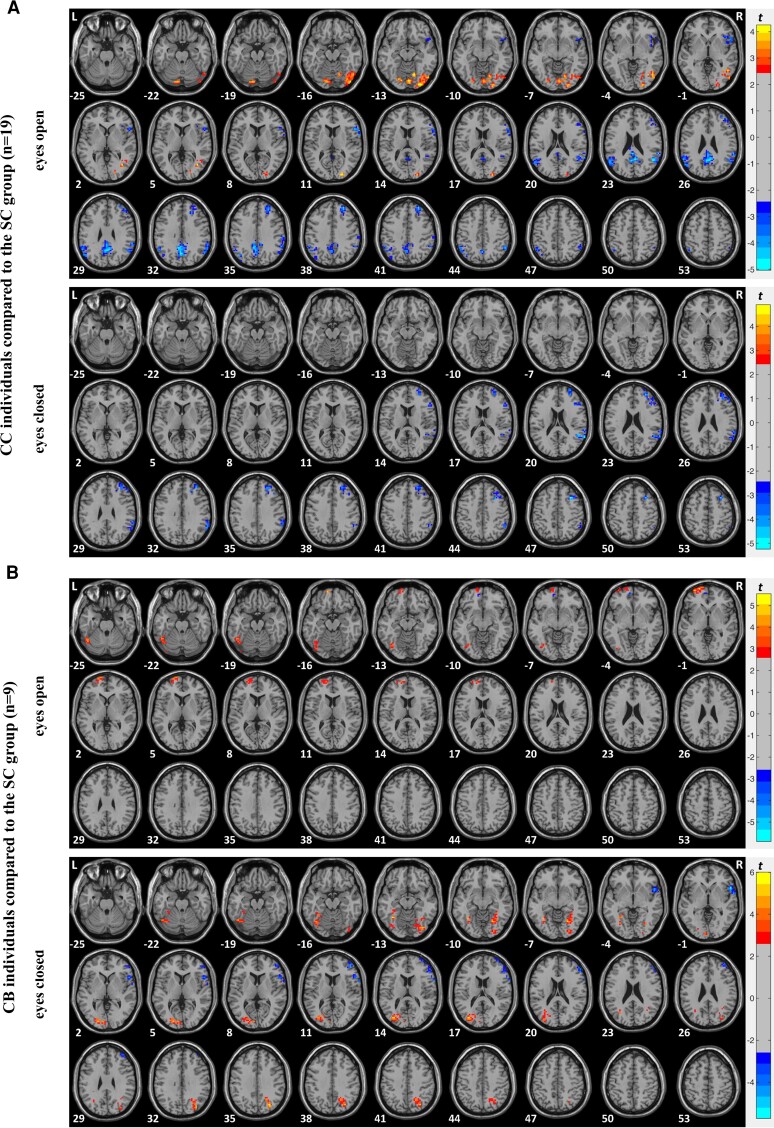
**ALFF in the EO and in the EC condition.** Two-sample *t*-test results of the ALFF comparing (**A**) congenital cataract-reversal individuals (CC) and the SC group (*n* = 19) matched in age and sex to the CC group and (**B**) congenitally blind individuals (CB) and the SC group (*n* = 9) matched in age and sex to the CB group in the eyes open (EO) condition and in the eyes closed (EC) condition. The red colours denote voxels with significantly higher amplitude for (**A**) the CC compared with the SC group and for (**B**) the CB compared with the SC group and the blue colours denote voxels with significantly lower amplitude for (**A**) the CC compared with the SC group and for (**B**) the CB compared with the SC group. Significant clusters are shown after GRF correction for multiple spatial comparisons (voxel-wise *P* < 0.01, cluster-wise *P* < 0.05, corrected).

Since eyes open versus closed does not mean the same for the CB group as for the other groups and to identify whether the CC group shows different resting-state activity particularly in the EO condition (see hypotheses), the CC group and their matched SC group were separately compared in the EO and in the EC condition using voxel-wise *t*-tests (Fig. 4A; Supplementary Table 3). The analogous analyses were run for the CB group and the DC group (Fig. 4B and Supplementary Figs. 2A and B; Supplementary Table 3). In addition, the CC group and the CB group were separately compared in the EO and in the EC condition using voxel wise *t*-tests (Supplementary Fig. 4; Supplementary Table 4). The statistical maps were corrected for multiple comparisons using a GRF correction (voxel-wise *P* < 0.01, cluster-wise *P* < 0.05, corrected).^74^

A probabilistic cytoarchitectonic atlas of the human brain (Automated Anatomical Labeling, Harvard-Oxford Cortical and Subcortical Structural Atlases, and Brodmann Atlas) as implemented in the DPABI toolbox, was used to assign significant voxels to brain regions.

### Data availability

Anonymized data and materials will be made available to the external scientists upon reasonable request to the corresponding author through data transfer agreements approved by the stakeholders, under stipulations of applicable law including but not limited to the General Data Protection Regulation (EU 2016/679).

## Results

### Amplitude of low-frequency fluctuation in the eyes open compared with the eyes closed condition in sighted control individuals (*n* = 28)

To determine brain regions with significantly higher and lower ALFF in the EO compared with the EC condition in sighted controls voxel-wise paired *t*-test was carried out (with correction for multiple comparisons see *Data analysis* section). We found significantly higher ALFF in a cluster in left visual association areas (BA7, 19), as well as in a cluster in the right visual association areas (BA7, 19) and in a cluster in the left precuneus (BA7). In addition, ALFF was higher in the EO than in the EC condition in a cluster in the left frontal cortex (BA8, 9, 46). ALFF was significantly lower in the EO than in the EC condition bilaterally in sensorimotor and temporal (auditory) regions i.e. in clusters covering precentral (BA4, 6) and postcentral gyrus (BA3), middle temporal gyrus, superior temporal gyrus and inferior temporal gyrus (BA20, 21, 22). In addition, ALFF was significantly lower in the EO than in the EC condition in a cluster in the left frontal regions (BA4, 8, 9) (see Supplementary Table 1 and Fig. 1 for more details).

In sum, in the SC group in the EO compared with the EC condition significantly higher ALFF was found in the visual association areas, parietal cortex and frontal regions, whereas lower ALFF was observed in sensorimotor and auditory regions. These results, by and large, replicated previously reported findings.^45–47,73^

### Amplitude of low-frequency fluctuation in the congenital cataract-reversal group versus the sighted control group (*n* = 19) in the eyes open versus the eyes closed condition

To determine brain regions with significantly higher and lower ALFF in congenital cataract-reversal individuals in the EO compared with the EC condition, voxel-wise paired *t*-test was carried out. We found significantly higher ALFF in the EO compared with the EC condition in a cluster in the right and left visual cortex including the calcarine gyrus (BA17), lingual gyrus (BA18) and the middle occipital gyrus (BA19). Significantly lower ALFF in the EO compared with the EC condition was found in a cluster in the left cingulate gyrus (BA23) and in a cluster in the supramarginal (BA40) and postcentral gyrus (BA2, 3) (see Fig. 2 and Supplementary Table 2).

To test for possible group differences, the interaction of group and condition was analyzed using a 2 × 2 mixed effect model (CC group versus SC group x EO condition versus EC condition) with the standardized ALFF maps (*Z*-scores) as the dependent variable. A significant interaction was found in a large cluster in right visual areas spanning the calcarine gyrus (BA17), lingual gyrus (BA18), middle occipital gyrus (BA19), and the fusiform gyrus (BA37) and in a cluster predominantly in the precuneus (BA7) (see Fig. 3 and Table 4 for more details).

We next compared the CC and the SC group separately in the EO and in the EC condition with voxel-wise two-sample *t*-tests. In the EO condition, we observed significantly higher ALFF in the CC group than in the SC group in a cluster in the right visual cortex including the calcarine gyrus (BA17), lingual gyrus (BA18) and the inferior and middle occipital gyrus (BA19). Significantly lower ALFF in the CC group than in the SC group was observed in a cluster in the right precuneus (BA7), and in clusters including parietal (BA39, 40), temporal (BA22) and frontal regions (BA8, 9, 45, 46) (see Supplementary Table 3; Fig. 4A upper panel: eyes open). In the EC condition, we did not observe any region with significantly higher ALFF in the CC group than in the SC group. Significantly lower ALFF in the CC group than in the SC group was observed in the right hemisphere in a cluster in frontal regions (BA8, 9, 46) and in a cluster in temporal (BA22) and parietal regions (BA39, 40) (see Supplementary Table 3 and Fig. 4A lower panel: eyes closed).

In sum in the CC group, we found higher ALFF in the EO as compared with the EC condition in visual cortex similar to the SC group. However, in the EO condition visual cortex activity was higher in the CC than in the SC group. In fact, the CC group showed an increase in ALFF in those early visual areas for which the normally sighted individuals typically show a decrease for the EO as compared with the EC condition. In addition, in the CC group, an increase of ALFF in parietal cortex was not observed in the EO compared with the EC condition and in fact, ALFF was lower here in the CC group than in the SC group. Moreover, in the CC group we did not observe the typical decrease of ALFF for the EO compared with the EC condition in auditory regions and in sensorimotor regions the decrease of ALFF in the EO as compared with EC was less robust in the CC group than in the SC group.

### Amplitude of low-frequency fluctuation in the developmental cataract-reversal group versus the sighted control group (*n* = 11) in the eyes open versus the eyes closed condition

To determine brain regions with significantly higher and lower ALFF in developmental cataract-reversal individuals in the EO compared with the EC condition voxel-wise paired *t*-test was carried out. Significantly higher ALFF in the EO compared to the EC condition in the DC group was observed bilaterally in several clusters of the frontal cortex (BA8, 9, 45, 46). Significantly lower ALFF in the EO compared with the EC condition was observed in a cluster in the right early visual areas i.e. in the calcarine gyrus (BA17) and lingual gyrus (BA18), and in a cluster in temporal regions (BA21). In addition, lower ALFF was found in a cluster in sensorimotor areas including the precentral (BA4) and postcentral gyrus (BA2, 3) (Supplementary Fig. 3B; Supplementary Table 2).

A 2 × 2 mixed effect model (DC group versus SC group x EO condition versus EC condition) was calculated with standardized ALFF maps (*Z*-scores) as the dependent variable to test the interaction between group and condition. A significant interaction was found in a cluster in parietal cortex (BA7) (Supplementary Fig. 1A; Table 4).

We next compared ALFF between the DC and the SC group separately in the EO and in the EC condition with voxel-wise two-sample t-tests. In the EO condition, we observed significantly lower ALFF in the DC group than in the SC group in a cluster in parietal cortex, predominantly in the precuneus (BA7). We did not observe any region with significantly higher ALFF in the DC group compared with the SC group (Supplementary Table 3; Supplementary Fig. 2A). In the EC condition we did not observe any region with significantly higher or lower ALFF in the DC compared with the SC group (Supplementary Fig. 2B).

In sum, ALFF patterns in the EO as compared with EC condition in the DC group resembled those of the SC group. Parietal cortex activity was lower in the DC than the SC group during EO.

### Amplitude of low-frequency fluctuation in the congenitally blind group versus the sighted control group (*n* = 9) in the eyes open versus the eyes closed condition

To determine brain regions with significantly higher and lower ALFF in congenitally blind individuals in the EO compared with the EC condition voxel-wise paired *t*-test was carried out. We found significantly higher ALFF in the EO compared with the EC condition in a cluster in frontal regions (BA8, 9, 46). Significantly lower ALFF in the EO compared with the EC condition was found in a cluster in parietal cortex (BA7) (Supplementary Fig. 3C; Supplementary Table 2).

To analyze the interaction of group and condition a 2 × 2 mixed effect model (CB group versus SC group x EO condition versus EC condition) was carried out with standardized ALFF (*Z*-scores) as the dependent variable. Significant interaction effects were observed in a cluster in parietal regions (BA7) and in a cluster in frontal regions (BA8, 9, 46) (Supplementary Fig. 1B; Table 4).

We then compared the CB group and SC group separately in the EO and in the EC condition with voxel-wise two-sample *t*-tests. In the EO condition, we found significantly higher ALFF in the CB group than in the SC group in a cluster in the left visual areas spanning the middle occipital gyrus (BA19) and fusiform gyrus (BA37) and in a cluster in frontal cortex (BA8, 9). We did not observe any regions with significantly lower ALFF for the CB group compared with the SC group (Supplementary Table 3; Fig. 4B upper panel: eyes open). In the EC condition, we observed significantly higher ALFF in the CB group than in the SC group in two clusters in the left visual cortex: one in the middle occipital gyrus (BA19) and one in the fusiform gyrus (BA37) and in two clusters in right visual areas: one in dorsal visual association cortex (BA7, 19) and one in ventral visual cortex (BA18, 37). Significantly lower ALFF in the CB group than in the SC group was found in the right hemisphere in two clusters: one in frontal regions (BA45, 46) and one in frontal-temporal regions (BA22, 45) (Supplementary Table 3; Fig. 4B lower panel: eyes closed).

In sum, we found overall higher ALFF in the CB than in SC individuals in a wide range of visual areas and parietal cortex, whereas ALFF was lower in the CB than the SC group in frontal cortex during EC.

### Amplitude of low-frequency fluctuation in the congenital cataract-reversal group versus the congenitally blind group in the eyes open versus the eyes closed condition

In an explorative analysis, we compared the CC and the CB group separately in the EO and in the EC condition with voxel-wise two-sample *t*-tests. In the EO condition, we observed significantly higher ALFF in the CC group than in the CB group in a cluster in the superior frontal gyrus (BA8, 9). Significantly lower ALFF in the CC group than in the CB group was found in two clusters in parietal cortex (BA39, 40) (see graphical abstract; Supplementary Table 4; Supplementary Fig. 4A). In the EC condition, we observed significantly lower ALFF in the CC group than in the CB group in the right hemisphere in a cluster located in early visual cortex and visual association areas spanning calcarine gyrus (BA17), lingual gyrus (BA18), middle occipital gyrus (BA19) and fusiform gyrus (BA37), and in the left hemisphere in two clusters: one in visual association areas spanning middle occipital gyrus (BA19), fusiform gyrus (BA37) and including parts of the cerebellum and one located in early visual areas and visual association areas spanning calcarine gyrus (BA17), lingual gyrus (BA18) and middle occipital gyrus (BA19). We did not observe any regions with significantly higher ALFF in the CC group than in the CB group (Supplementary Table 4; Supplementary Fig. 4B).

In sum, compared with the CB group, CC individuals showed lower ALFF in a wide range of visual cortical regions in the EC condition.

## Discussion

The goal of the present study was to identify whether the emergence of typical resting-state activity of the human brain, as the prerequisite of any task related processing, depends on experience during a sensitive period of early brain development. To this end, we tested to which degree BOLD resting-state activity in the low frequency range between 0.01 and 0.08 Hz during EO and EC recovered after a transient phase of congenital blindness due to congenital cataracts. Congenital cataract-reversal individuals (CC group) were compared with normally sighted controls (SC group) and to a group of congenitally blind humans (CB group). Developmental cataract-reversal individuals (DC group) served as additional control group. All groups were investigated in the same scanner, came from the same community and the groups were matched in age.

First, we replicated the typical ALFF pattern in the SC group^45–47,73^: ALFF was significantly higher in the EO than in the EC condition in visual association cortex and in parietal cortex. Moreover, ALFF was significantly lower in the EO than in the EC condition in sensorimotor and auditory cortices.

Importantly, ALFF varied with EO versus EC in the CC group’s visual cortices as well: Similar to the SC group the amplitude of slow BOLD fluctuations was higher in the EO than in the EC condition. However, there were several group differences too: In the CC group’s early visual cortex, we did not observe a typical decrease of ALFF in the EO compared with the EC condition, instead ALFF was higher in this region in the EO than in the EC condition too. Moreover, in the EO condition visual cortex activity was overall higher in the CC than in the SC group. Furthermore, in the CC group an increase of ALFF in parietal cortex was not observed in the EO compared with the EC condition and thus was lower in the EO condition than in the SC group. Moreover, the typical decrease of ALFF for the EO compared with the EC condition was missing in auditory regions in the CC group and in sensorimotor regions the decrease was less extensive in the CC group than in the SC group. Finally, the CB group showed higher ALFF than SC individuals in both the EO and the EC condition, and higher ALFF than the CC group in the EC condition (for a graphic summary of the main results see the graphical abstract).

Research in non-human primates showed that synaptic pruning in visual cortex is experience dependent and particularly affects the asymmetric, excitatory synapses, resulting in an experience dependent set-point for visual cortex excitability. Cortical thickness development runs parallel to the developmental trajectory of synaptogenesis.^78^ In fact, permanently congenitally blind individuals feature thicker visual cortices which was interpreted as indicating an arrest of experience dependent synaptic pruning.^79–83^ Importantly, a higher cortical thickness has recently been observed in CC individuals^84,85^ too. These results thus suggest that the process of synaptic pruning in early visual cortex is linked to a sensitive period in early primate brain development. The presence of exuberant synapses has been demonstrated to result in higher glucose uptake^7,86^ and presumably blood flow during rest.^87^ Thus, we speculate that higher ALFF might reflect higher resting-state excitatory activity of less pruned neural circuits within the occipital lobe of CC (and CB) individuals.

Early visual cortex in sighted individuals is characterized by a high degree of inhibition, which results in a short time constant and thus the ability to process visual information at a fast rate.^88^ Non-human animal research has demonstrated that the elaboration of inhibitory neural networks is a hallmark of sensitive period plasticity.^89^ In fact, stabilization of inhibitory synapse and myelination ends the sensitive period.^90^

Previous EEG studies in CB and CC individuals have repeatedly observed lower alpha oscillatory activity^53–57^ and in CB individuals higher gamma oscillatory activity.^58,59^ Alpha oscillatory activity has been considered to be an electrophysiological signature for the control of the excitatory–inhibitory (E/I) balance of neural circuits.^91^ In the present context, it is important to note that alpha oscillatory activity has been found to inversely correlate with ALFF^48,49^ whereas gamma band activity was found in monkeys to positively correlate with slow BOLD fluctuations.^62^ Thus, the higher posterior ALFF observed for the CC and the CB groups in the present study is consistent with reduced posterior alpha band activity in these groups and higher gamma band activity reported for CB individuals. All these findings converge to the hypothesis that overall excitation is enhanced in the visual cortex of CB and CC individuals. Moreover, the visually triggered BOLD signal seems to be correlated with changes in the glutamate level.^92^ In fact, there is evidence of higher glutamatergic^93^ and lower GABAergic activity in congenitally permanently blind individuals.^94^ Corresponding data in CC individuals are not available yet.

However, there was a crucial difference between the CC and the CB group. Higher ALFF was observed for the CC group than in the SC group only in the EO condition and in fact in the EC condition ALFF was lower in the CC than in the CB group. These group differences demonstrate that the visual cortical networks partially recovered in the CC group, that is, different resting-state activity levels were adopted as a function of whether or not light reaches the retina during rest.

Visual thalamo-cortical input excites pyramidal neurons in the granular layers of the cortex but in parallel entertains synapses to inhibitory interneurons, which allows a quick shutting down of excitation. From non-human animal research, it is known that these inhibitory circuits are shaped by experience and that they are stabilized by perineural networks^89^ resulting in neural circuits^95^ which selectively respond only to certain input. Thus, we speculate that higher ALFF in the CC group in the EO condition indicates less selective processing and an impaired quick shutting down of visual-driven activation possibly due to a compromised intracortical inhibition. In fact, behavioural studies have shown longer lasting visual (motion) after-effects in CC individuals,^22^ which indirectly supports this speculation. By contrast, we hypothesize that higher ALFF in visual cortex in the CB group was predominantly due to higher spontaneous activity. Higher spontaneous activity in visual areas is an often reported finding in visually deprived non-human animals.^96,97^ In fact, a study in monkeys has observed a decrease of spontaneous activity in visual cortex after ending a phase of congenital lid suture.^96^ Thus, a decrease in spontaneous activity might explain the lower ALFF in visual areas in the CC than in the CB group during the EC condition and might explain as well indistinguishable ALFF of the CC and the SC groups in the EC condition. Lower spontaneous activity would be compatible with the idea of partial E/I balance recovery in visual cortex.

In sum, we suggest that higher slow BOLD fluctuations in CB and CC individuals might originate from a similar neural substrate, that is, not or less well attuned visual circuits. However, despite late availability of patterned visual input, the neural circuits in the CC group seem to had recovered to some degree too, such that spontaneous activity decreased. However, the fine-tuned neural (inhibitory) circuits which allow for a selective activation and quick shutting down of stimulus-driven activity might not have fully emerged, resulting in an enhanced and possibly longer-lasting excitation as a response to visual stimulation.

Importantly, we interpret our results on slow BOLD fluctuations in the visual cortices of CC individuals as evidence for retracted cross-modal plasticity. ALFF in visual cortex was lower in the CC than in CB individuals and varied as a function of EO versus EC. In fact, in the context of cross-modal plasticity in deaf cats it has been argued that the higher level of excitation in their auditory cortex reflects a largely reduced threshold to allow for cross-modal activation.^8^ Although sensorineural deafness might cause more extensive changes in E/I balance than visual deprivation due to cataracts which do not directly affect the sensorineural architecture, the same mechanism might contribute to cross-modal plasticity in blind humans. Here we argue that, visual entrainment of visual areas in the CC group might have enhanced the threshold for cross-modal activation and reduced spontaneous activity possibly via homeostatic plasticity mechanisms resulting in lower ALFF in the CC group compared with the CB group during EC.^98^

In parietal cortex, we observed a lower ALFF in the CC than in the SC group in the EO condition. Hyvärinen *et al.*^99^ reported a lower visual responsiveness in parietal area BA7 in monkeys who had been visually deprived for 7-11 months. In a follow-up study 1 year after the end of the deprivation period responsiveness to visual stimulation had further declined rather than increased, as would have been expected from restoring sight.^100^ Except one participant (assessed 6 months post-cataract removal surgery), all CC participants of the present study were scanned more than 1 year after cataract removal surgery. Here, we speculate that the observed lower resting-state activity in the CC individuals in parietal regions during EO might reflect a similar reduced regain of visual responsiveness in parietal cortex as observed by Hyvärinen *et al.*^99^ in non-human primates. Although parietal cortex is a multisensory region, many processes including multisensory spatial integration are visually dominated in sighted individuals. Thus, lower ALFF in parietal cortex in the CC compared with the SC group might indicate a lower visual influence on multisensory (spatial) processing. Recently, Smyre *et al.*^101^ reported an impaired multisensory detection and localization in dark reared cats. In fact, two behavioural studies have found a reduced visual impact on tactile spatial performance in congenital cataract-reversal individuals with a history of longer lasting visual deprivation.^102,103^

Altered multisensory processing is suggested by the third main result in the CC group too: Activity in auditory regions was, in contrast to the SC group (and the DC group) not lower during EO than during EC and in sensorimotor regions the decrease of ALFF in the EO as compared with EC was less robust in the CC group that is rather small compared with the SC group (see Fig. 2). Functional connectivity studies in sighted humans have provided ample evidence for a higher functional coupling of visual and auditory, as well as visual and sensorimotor cortices during EC than during EO.^104^ Crucially, such overall coupling between visual brain regions and both auditory and sensorimotor brain regions seems to be reduced in congenitally blind humans.^66,67^ Our new finding that resting-state activity in auditory regions is unaffected and in sensorimotor regions less affected by eyes opening in CC individuals might suggest, analogously to parietal cortex, a reduced impact of vision in multisensory processing. This idea is compatible with the previously reported lower lip reading specific activity in the superior temporal sulcus of CC individuals^105^ and the lack of audio–visual enhancements neither in this region^18^ nor in behaviour.^106^

Finally, it has to be noted that similar group differences as found between the CC and the SC group were not observed for the DC group except the lower parietal cortex activity during EO. By contrast, the typical decrease in ALFF for resting-state activity with EO versus EC was highly robust in the DC group. Thus, a typical modulation of auditory and sensorimotor cortex activity by the visual system might crucially depend on connectivity elaborated in early brain development. By contrast, the lower impact of vision on parietal cortex activity condition might reflect a lower online weighting due to overall reduced reliability of visual input.^107^

## Conclusion

Slow BOLD fluctuations indicating resting-state activity of neural circuits suggested a retraction of cross-modal plasticity after sight restoration in individuals with a history of blindness due to congenital cataracts. However, visual neural circuits seemed to be less tuned and activity thus was not as well-regulated as in normally sighted controls. This impairment might reflect remaining visual cortical circuit changes as a consequence of congenital blindness.

A significant influence of the visual system on parietal areas, as well as the auditory system as typically found in sighted individuals had not recovered and in sensorimotor cortex not fully recovered in the congenital cataract-reversal individuals, suggesting a high influence of early experience on multisensory neural networks.

Since resting-state brain activity builds the scaffold for task-related processing, we put forward the hypothesis that the incomplete recovery of typical resting-state activity patterns within the visual system and across sensory systems might contribute to the persisting visual and multisensory impairments after restoring sight in people with a congenital loss of pattern vision.

## Supplementary Material

fcac146_Supplementary_Data

## Abbreviations

BA =: Brodmann area
CB =: congenitally blind individuals
CC =: congenital cataract-reversal individuals
DC =: developmental cataract-reversal individuals
EC =: eyes closed
EO =: eyes open
EPI =: echo-planar imaging
FA =: flip angle
fMRI =: functional magnetic resonance imaging
logMAR =: Logarithm of the Minimum Angle of Resolution
SC =: sighted control individuals
TE =: echo time
TR =: repetition time
