# Supplementary material for: Typical resting-state activity of the brain requires visual input during an early sensitive period
Source: Brain Commun. 2022 Jun 7;4(4):fcac146. doi: 10.1093/braincomms/fcac146 (PMC9275761; doi:10.1093/braincomms/fcac146)
Supplement: fcac146_Supplementary_Data [file fcac146_supplementary_data.pdf]

Supplementary Materials

Typical resting state activity of the brain requires visual input during an early sensitive period

Katarzyna Rączy[1], Cordula Hölig[1], Maria J. S. Guerreiro[1,2], Sunitha Lingareddy[3], Ramesh Kekunnaya[4], Brigitte Röder[1]

Author affiliations:

1 Biological Psychology and Neuropsychology, University of Hamburg, 20146 Hamburg, Germany

2 Biological Psychology, Department of Psychology, Carl Von Ossietzky University of Oldenburg, 26111, Oldenburg, Germany

3 Lucid Medical Diagnostics Banjara Hills, 500034 Hyderabad, India

4 Jasti V Ramanamma Children's Eye Care Center, Child Sight Institute, LV Prasad Eye Institute, 500034 Hyderabad, India

Correspondence to: Dr. Katarzyna Rączy

Biological Psychology and Neuropsychology

University of Hamburg

Von-Melle-Park 11

20146 Hamburg, Germany

email: katarzyna.raczy@uni-hamburg.de

Running title: Resting state activity in sight recovery

Supplementary Table 1. Brain regions showing differences in ALFF in the EO compared to the EC condition in sighted control individuals (SC group. n=28).

Hemisphere	Brain region	Brodmann area	Cluster size	Peak voxel MNI coordinates			Peak voxel t-value
				x	y	z	
L	Superior Temporal Gyrus / Postcentral Gyrus / Precentral Gyrus	2, 3, 4, 6 22	537	-52	-16	12	-4.91
L	Superior Parietal Gyrus / Middle Occipital Gyrus	7, 19	322	-18	-68	48	5.91
R	Middle Occipital Gyrus / Superior Occipital Gyrus / Superior Parietal Gyrus	7, 19	235	41	-78	20	5.44
R	Postcentral Gyrus / Precentral Gyrus	2, 3, 4	180	41	-6	12	-4.86
L	Precuneus / Cingulate Gyrus	7, 23	151	-1	-54	24	5.11
L	Precentral Gyrus / Superior Frontal Gyrus	4, 8, 9	133	-18	-19	80	-4.65
R	Middle Temporal Gyrus / Inferior Temporal Gyrus	20, 21	80	48	-64	-4	-5.11
L	Superior Frontal Gyrus / Middle Frontal Gyrus	8, 9, 46	62	-18	56	12	4.19

Note. Clusters showing group differences in ALFF between the EO and the EC condition at $p < .01$ voxel-wise and $p < .05$ cluster-wise after Gaussian random field (GRF) correction for multiple comparisons. MNI coordinates and t-values are derived from the peak voxel of the cluster. A positive t-value means greater ALFF in the EO condition and a negative t-value means greater ALFF in the EC condition. EO = eyes open. EC = eyes closed. MNI = Montreal Neurological Institute coordinates system. L = left. R = right.

Supplementary Table 2. Brain regions showing differences in ALFF in the EO compared to the EC condition for each group.

Hemisphere	Brain region	Brodmann areas	Cluster size	Peak voxel MNI coordinates			Peak voxel t-value
				x	y	z	
**ALFF in the SC group (n=19) in the EO vs. the EC condition**							
L	Postcentral Gyrus / Precentral Gyrus	2, 3, 4	193	-49	-23	40	-5.44
L	Medial Frontal Gyrus / Superior Frontal Gyrus / Middle Frontal Gyrus	8, 9, 46	122	-4	53	8	4.67
R	Supramarginal Gyrus / Postcentral Gyrus / Precentral Gyrus	2, 3, 4, 40	79	48	-26	36	-4.40
R	Middle Temporal Gyrus / Middle Occipital Gyrus / Inferior Temporal Gyrus	19, 20, 21	72	48	-64	-4	-5.40
R	Cingulate Gyrus / Medial Frontal Gyrus / Supplementary Motor Area	6, 8, 9, 23	70	13	-13	48	-3.78
R	Medial Frontal Gyrus / Superior Frontal Gyrus	8, 9	57	6	-13	80	-3.71
L	Superior Parietal Gyrus / Precuneus	7	55	-18	-68	48	5.04
L	Precuneus / Cingulate Gyrus	7, 23	49	-11	-50	24	3.40
**ALFF in the CC group (n=19) in the EO vs. the EC condition**							
L	Calcarine Gyrus / Lingual Gyrus / Middle Occipital Gyrus	17, 18, 19	359	-28	-85	16	4.62
L	Cingulate Gyrus	23	102	-4	-44	16	-4.51
R	Postcentral Gyrus / Supramarginal Gyrus	2, 3, 40	50	48	-30	56	-4.28
**ALFF in the DC group (n=11) in the EO vs. the EC condition**							
R	Precentral Gyrus / Postcentral Gyrus / Superior Temporal Gyrus / Supplementary Motor Area / Cingulate Gyrus	2, 3, 4, 6, 22, 23	1018	44	-13	16	-7.20

R	Middle Frontal Gyrus / Inferior Frontal Gyrus / Superior Frontal Gyrus	8, 9, 45, 46	311	44	25	0	6.97
L	Superior Frontal Gyrus	8, 9	58	-18	39	48	4.38
L	Superior Frontal Gyrus / Middle Frontal Gyrus	8, 9, 46	55	-18	67	16	5.38
R	Superior Frontal Gyrus / Middle Frontal Gyrus	8, 9, 46	50	13	39	40	4.92
R	Middle Temporal Gyrus	21	46	41	-57	-12	-5.01
R	Calcarine Gyrus / Lingual Gyrus	17, 18	44	23	-47	-4	-5.34
**ALFF in the CB group (n=9) in the EO vs. the EC condition**							
L	Superior Frontal Gyrus / Middle Frontal Gyrus / Medial Frontal Gyrus	8, 9, 46	679	-38	60	0	12.99
L	Precuneus / Superior Parietal Gyrus	7	45	-18	-71	40	-5.37

Note. Clusters showing group differences in ALFF between the EO and the EC condition at $p < .01$ voxel-wise and $p < .05$ cluster-wise after Gaussian random field (GRF) correction for multiple comparisons. MNI coordinates and t-values are derived from the peak voxel of the cluster. A positive t-value means greater ALFF in the EO condition and a negative t-value means greater ALFF in the EC condition. EO = eyes open. EC = eyes closed. MNI = Montreal Neurological Institute coordinates system. L = left. R = right.

Supplementary Table 3. Brain regions showing differences in ALFF between the tested groups separately in the EO and in the EC condition.

Hemisphere	Brain region	Brodmann area	Cluster size	Peak voxel MNI coordinates			Peak voxel t-value
				x	y	z	
**ALFF in the CC group (n=19) vs. SC group (n=19)**							
**in the EO condition**							
R	Calcarine Gyrus / Lingual Gyrus / Middle Occipital Gyrus / Inferior Occipital Gyrus	17, 18, 19	286	30	-92	-12	4.26
R	Precuneus / Cingulate Gyrus	7, 23	195	6	-54	24	-5.04
R	Inferior Frontal Gyrus / Superior Frontal Gyrus / Middle Frontal Gyrus	8, 9, 45, 46	137	27	46	40	-4.48
L	Superior Temporal Gyrus / Angular Gyrus / Supramarginal Gyrus	22, 39, 40	126	-56	-61	24	-3.78
R	Superior Temporal Gyrus / Angular Gyrus / Supramarginal Gyrus	22, 39, 40	115	48	-54	24	-4.53
**in the EC condition**							
R	Middle Frontal Gyrus / Superior Frontal Gyrus	8, 9, 46	153	30	18	48	-5.05
R	Supramarginal Gyrus / Angular Gyrus / Superior Temporal Gyrus	22, 39, 40	91	65	-50	20	-4.58
**ALFF in the DC group (n=11) vs. SC group (n=11)**							
**in the EO condition**							
L	Precuneus / Cingulate Gyrus	7, 23	96	-8	-61	28	-5.40
**ALFF in the CB group (n=9) vs. SC group (n=9)**							
**in the EO condition**							
L	Superior Frontal Gyrus / Medial Frontal Gyrus	8, 9	104	-32	63	0	5.03
L	Middle Occipital Gyrus / Fusiform Gyrus	19, 37	51	-45	-54	-20	3.96
**in the EC condition**							

L	Middle Occipital Gyrus	19	103	-35	-78	16	4.97
R	Precuneus / Superior Occipital Gyrus / Middle Occipital Gyrus	7, 19	79	30	-71	36	6.01
R	Lingual Gyrus / Fusiform Gyrus	18, 37	68	23	-64	-8	4.78
R	Inferior Frontal Gyrus / Superior Temporal Gyrus	22, 45	65	54	18	0	-5.49
L	Fusiform Gyrus	37	62	-35	-54	-12	5.51
R	Middle Frontal Gyrus / Inferior Frontal Gyrus	45, 46	58	44	49	12	-4.00

Note. Clusters showing group differences in ALFF between the tested groups separately in the EO and in the EC condition at $p < .01$ voxel-wise and $p < .05$ cluster-wise after Gaussian random field (GRF) correction for multiple comparisons. MNI coordinates and t-values are derived from the peak voxel of the cluster. A positive t-value means greater ALFF in the CC group, the DC group and the CB group, respectively, and a negative t-value means greater ALFF in the SC group. EO = eyes open. EC = eyes closed. MNI = Montreal Neurological Institute coordinates system. L = left. R = right.

Supplementary Table 4. Brain regions showing differences in ALFF between the CC individuals (n=19) and CB individuals (n=9) separately in the EO and in the EC condition. .

Hemisphere	Brain region	Brodmann area	Cluster size	Peak voxel MNI coordinates			Peak voxel t-value
				x	y	z	
**in the EO condition**							
L	Superior Frontal Gyrus [a]	8, 9	154	-8	1	72	3.48
L	Supramarginal Gyrus / Angular Gyrus	39, 40	76	-56	-54	28	-4.35
R	Supramarginal Gyrus	40	65	61	-40	32	-3.75
**in the EC condition**							
R	Calcarine Gyrus / Lingual Gyrus / Middle Occipital Gyrus / Fusiform Gyrus	17, 18, 19, 37	221	30	-71	-4	-4.95
L	Middle Occipital Gyrus / Fusiform Gyrus / Cerebellum	19, 37	157	-35	-64	-20	-6.21
L	Calcarine Gyrus / Lingual Gyrus / Middle Occipital Gyrus	17, 18, 19	132	-35	-81	16	-4.48

Note. Clusters showing group differences in ALFF between the CC individuals (n=19) and CB individuals (n=9) in the EC condition at $p < .01$ voxel-wise and $p < .05$ cluster-wise after Gaussian random field (GRF) correction for multiple comparisons. MNI coordinates and t-values are derived from the peak voxel of the cluster. A positive t-value means greater ALFF in the CC group and a negative t-value means greater ALFF in the CB group. EO = eyes open. EC = eyes closed. MNI = Montreal Neurological Institute coordinates system. L = left. R = right.

[a] This cluster is not visible in the Supplementary Figure 4A.

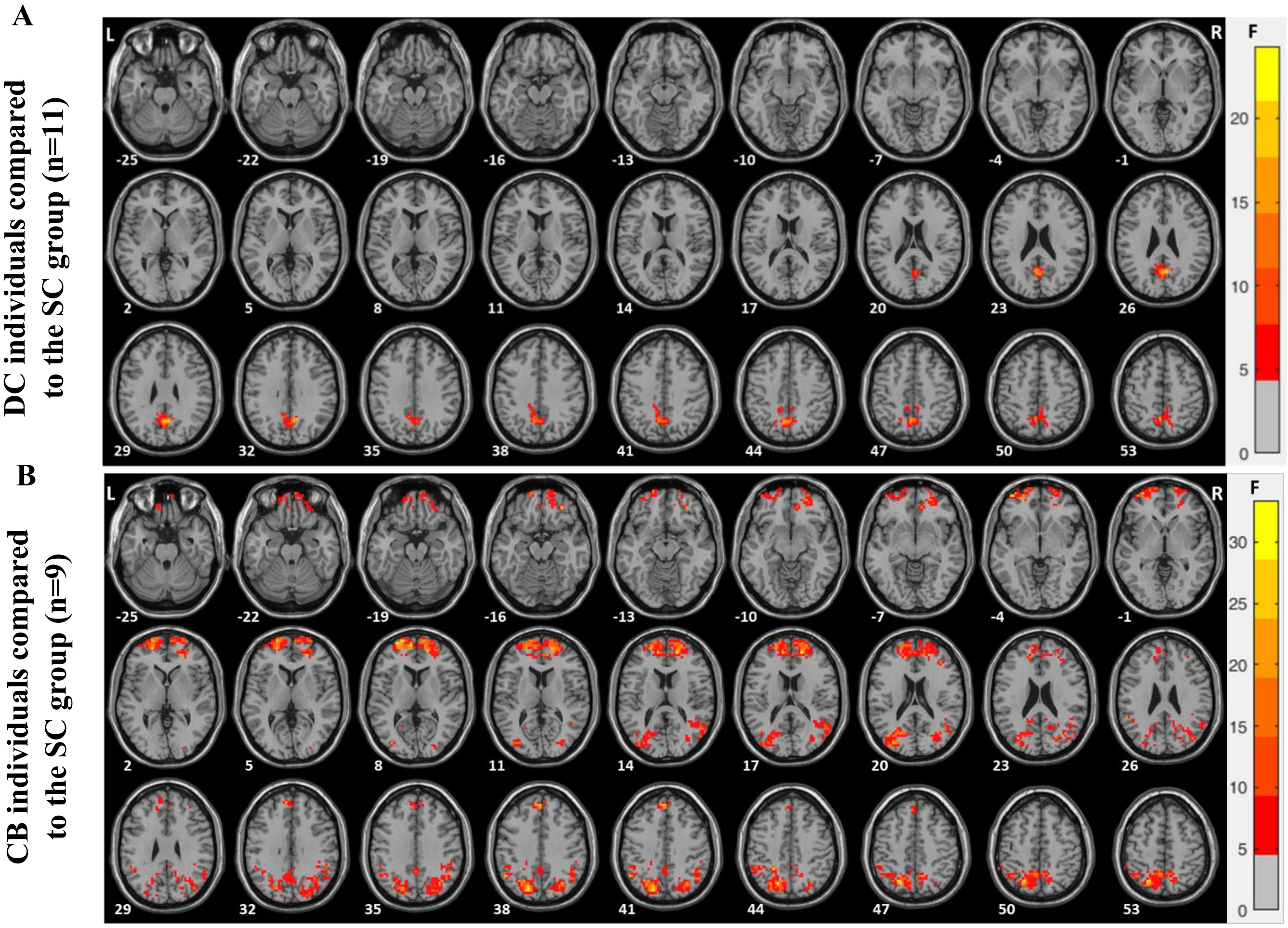

Supplementary Figure 1 Group differences in ALFF in the EO compared to the EC condition. Using standardized ALFF (amplitude of low frequency fluctuations) i.e. Z-scores, a mixed 2x2 model (group x condition) was carried out for **(A)** developmental cataract-reversal individuals (DC) vs. SC group (n=11) and **(B)** congenitally blind individuals (CB) vs. SC group (n=9). Regions with significant interaction effects are shown after Gaussian random field theory (GRF) correction for multiple spatial comparisons (voxel-wise $p < .05$, cluster-wise $p < .025$, corrected).

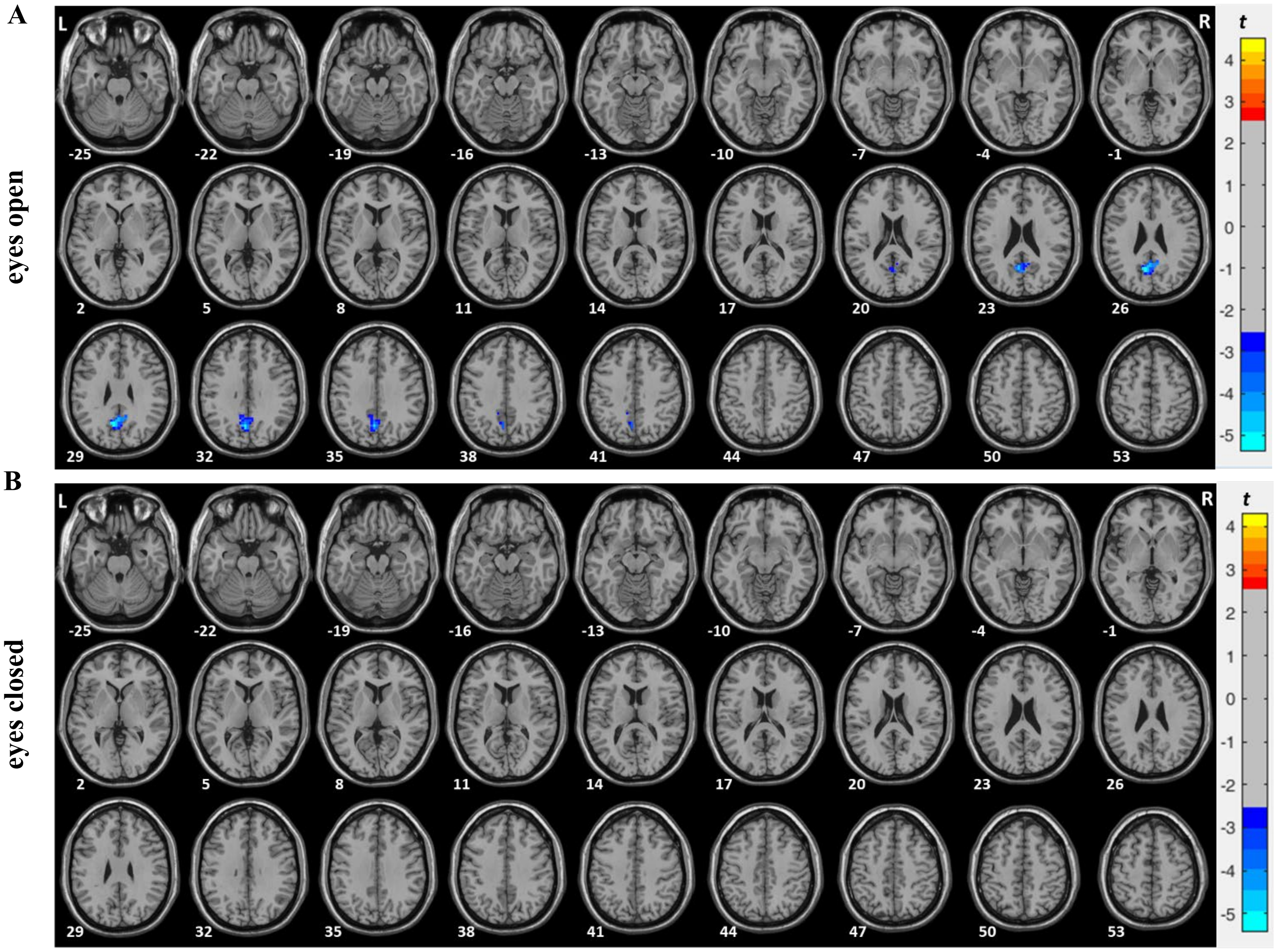

Supplementary Figure 2 ALFF in the DC group (n=11) compared to the SC group (n=11) in the EO and in the EC condition. Two-sample t-test results of the amplitude of low frequency fluctuations (ALFF) comparing developmental cataract-reversal individuals (DC) and the SC group (n=11) matched in age and sex to the DC group separately in the **(A)** eyes open (EO) condition and in the **(B)** eyes closed (EC) condition. The red colors denote voxels with significantly higher amplitude for the DC compared to the SC group and the blue colors denote voxels with significantly lower amplitude for the DC compared to the SC group. Significant clusters are shown after Gaussian random field theory (GRF) correction for multiple spatial comparisons (voxel-wise $p < .01$, cluster-wise $p < .05$, corrected).

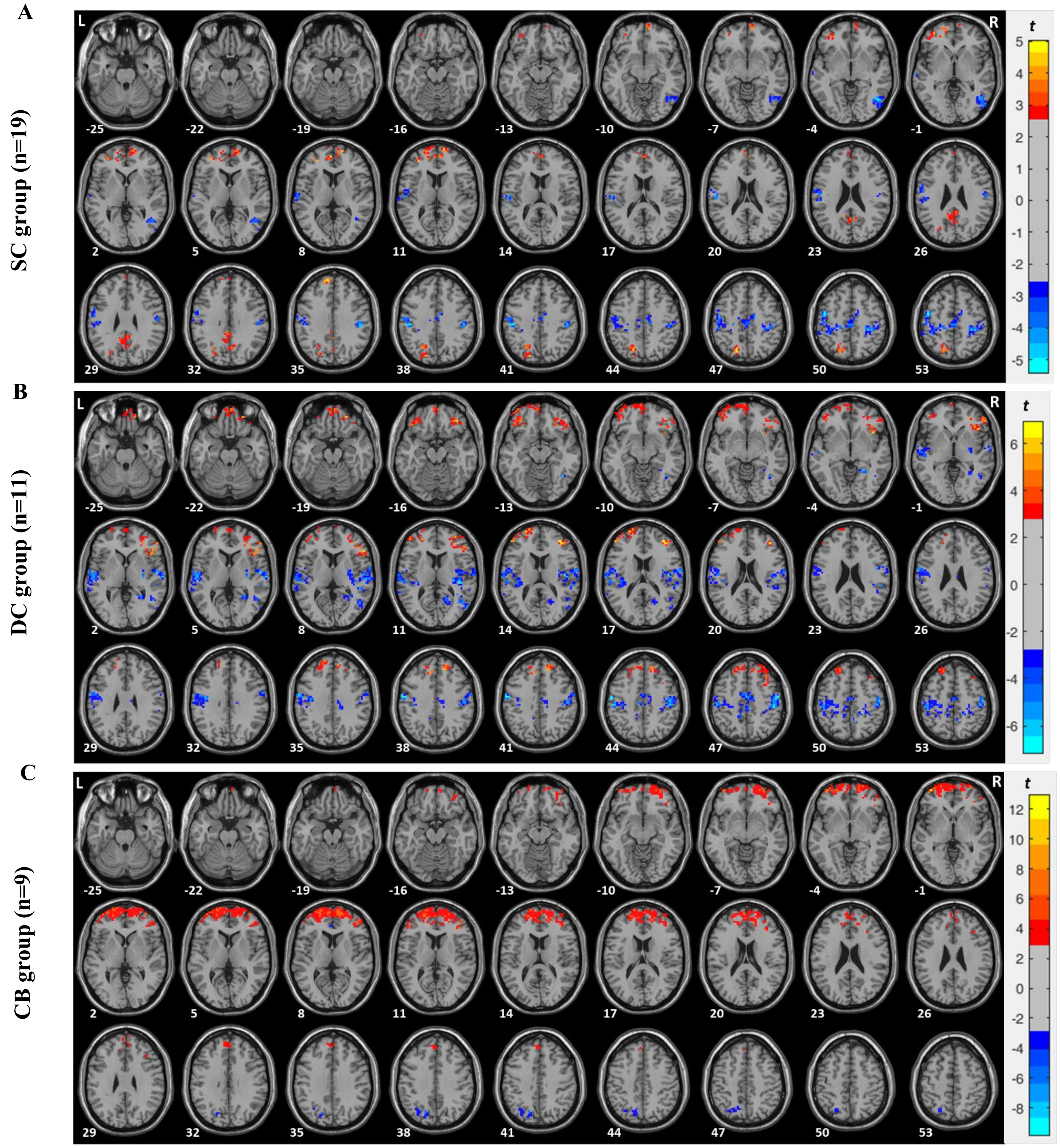

Supplementary Figure 3 ALFF in the EO compared to the EC condition Paired t-test results of the amplitude of low frequency fluctuations (ALFF) comparing the eyes open (EO) and the eyes closed (EC) condition in the group of **(A)** sighted individuals (n=19) matched in age and sex to CC individuals **(B)** developmental cataract-reversal individuals and **(C)** congenitally blind individuals. The red colors denote voxels with significantly higher amplitude for the EO compared to the EC condition and the blue colors denote voxels with significantly lower amplitude for the EO compared to the EC condition. Significant clusters are shown after Gaussian random field (GRF) correction for multiple spatial comparisons (voxel-wise $p < .01$, cluster-wise $p < .05$, corrected).

A

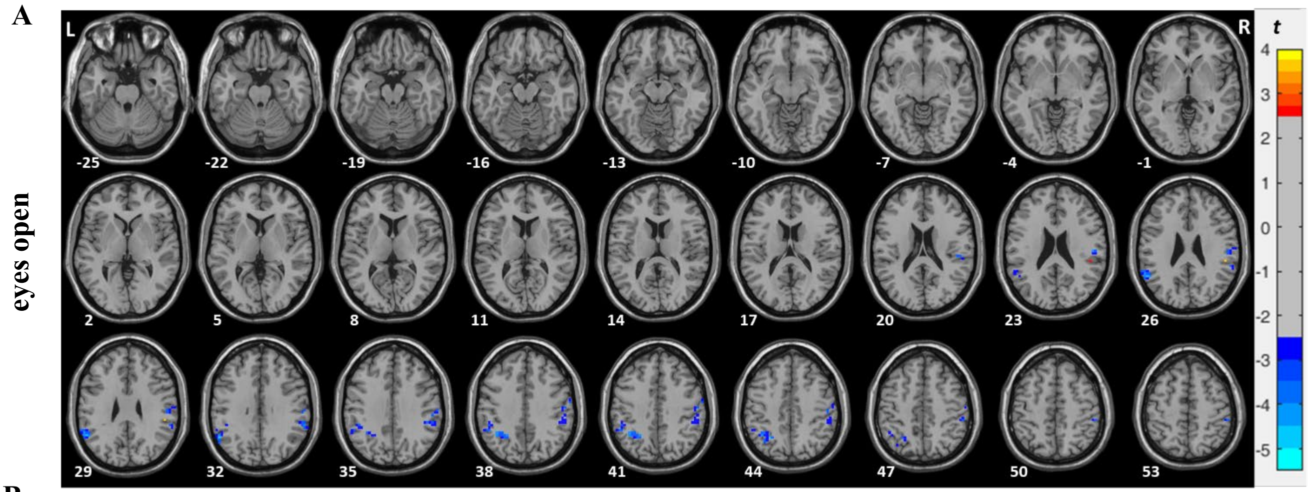

B

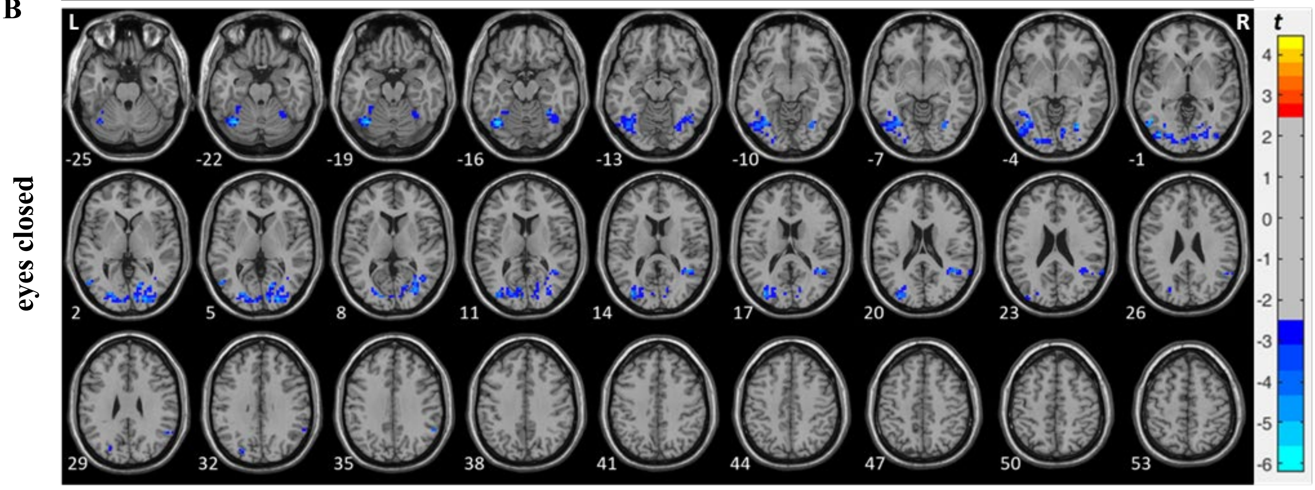

Supplementary Figure 4 ALFF in the CC group (n=19) compared to the CB group (n=9) in the EO and in the EC condition. Two-sample t-test results of the amplitude of low frequency fluctuations (ALFF) comparing congenital cataract-reversal individuals (CC group, n=19) and congenitally blind individuals (CB group, n=9) in the **(A)** eyes open (EO) and in the **(B)** eyes closed (EC) condition. The red colors denote voxels with significantly higher amplitude for the CC group compared to the CB group and the blue colors denote voxels with significantly lower amplitude for the CC group compared to the CB group. Significant clusters are shown after Gaussian random field theory (GRF) correction for multiple spatial comparisons (voxel-wise $p < .01$, cluster-wise $p < .05$, corrected).
